# Mpox [em′poks] and Monkeypox Virus [muhng′kee poks′ vī-ruhs′]

**DOI:** 10.3201/eid3108.240211

**Published:** 2025-08

**Authors:** Clyde Partin

**Affiliations:** Emory University, Atlanta, Georgia, USA

**Keywords:** Mpox, monkeypox virus, MPXV, viruses, zoonoses

**Figure 1:**
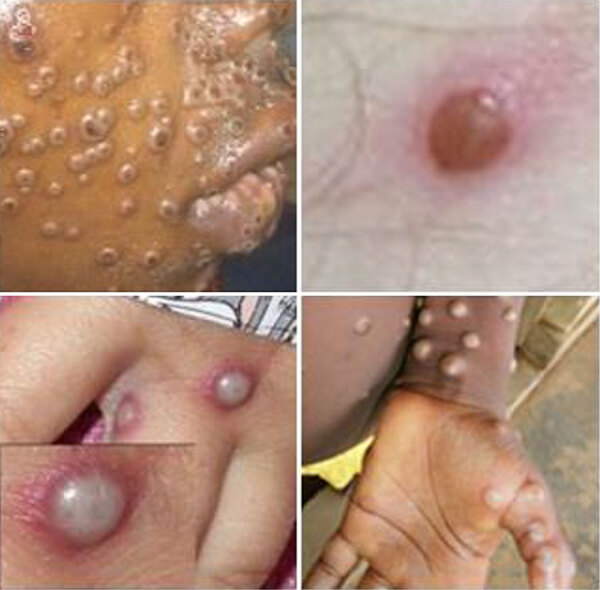
Examples of pustular eruptions from monkeypox virus. Image source: Centers for Disease Control and Prevention (https://www.cdc.gov/mpox/hcp/clinical-signs).

**Figure 2:**
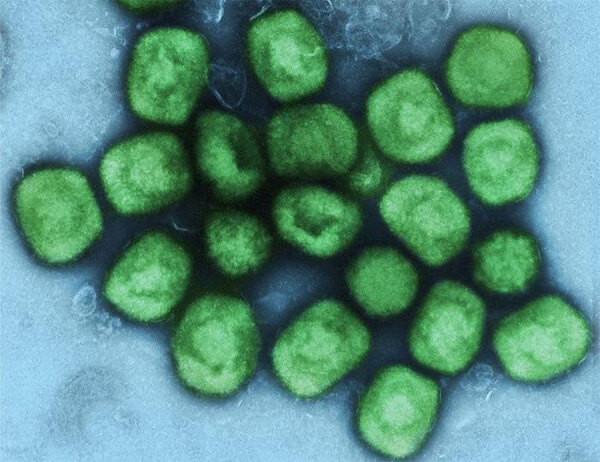
Colorized transmission electron microscopic image of monkeypox virus particles (green) that were cultivated and purified from cell culture. The image was captured at the National Institute of Allergy and Infectious Diseases Integrated Research Facility, Fort Detrick, Maryland, USA.

Mpox, the infectious disease caused by monkeypox virus (MPXV), is characterized in humans by fever, lymphadenopathy, and a painful mucocutaneous rash. During 1958–1959, Danish virologist Preben von Magnus isolated the virus in macaque monkeys—hence, the name monkeypox (Figures 1,2). However, small mammals are likely the MPXV reservoir, not the aberrantly infected monkeys that received unwarranted blame as the host and created the misnomer monkeypox.

The derivation of the word monkey is shrouded in debate, antiquity, and complexity. The Oxford English Dictionary postulates *Moneke* referred to the son of Martin the Ape, a character in a collection of beloved European fables, Reynard the Fox, and the moniker might have accompanied continental showmen to England in the 1400s, but earlier variations of *Moneke* exist in other languages.

Pox, plural of pock, is Norman-French in origin from *poque*, meaning pouch. The diminutive form was *poquet*, or little pocket, describing the small scars remaining in the wake of various viral pustular eruptions, classically smallpox or syphilis. Plague and pox appear interchangeably in Shakespeare’s 1597 play Romeo and Juliet, in which Mercutio utters the famous and pejorative phrase, “A pox on both your houses.”

The first known human case of MPXV infection was reported in the Democratic Republic of the Congo in 1970. A global outbreak in 2022 was the first that involved widespread human-to-human MPXV transmission outside of Africa, prompting the World Health Organization to declare a Public Health Emergency. In November 2022, citing concerns of “racist and stigmatizing language” provoked by the original disease name, the World Health Organization renamed MPXV infection as mpox, but the virus name remains unchanged.
